# Study of Some Inflammatory Mediators in the Serum of Patients With Atherosclerosis and Acute Myocardial Infarction

**DOI:** 10.7759/cureus.18450

**Published:** 2021-10-03

**Authors:** Ohoud Metwalli, Enayat Hashem, Mohammed Ali Ajabnoor, Nabil Alama, Zainy M Banjar

**Affiliations:** 1 Pathology, Department of Laboratory, King Abdulaziz University Hospital, Jeddah, SAU; 2 Clinical Biochemistry, King Abdulaziz University Faculty of Medicine, Jeddah, SAU; 3 Cardiology, Department of Medicine, King Abdulaziz University Hospital, Jeddah, SAU

**Keywords:** myocardial infarction, resistin, serum amyloid a, adiponectin, atherosclerosis

## Abstract

Background and aim of the study

The aim of this study is to evaluate the changes in the inflammatory mediator’s serum amyloid A (SAA), adiponectin, and resistin in the serum of patients with stable angina and acute myocardial infarction.

Subjects and methods

The study was done on 60 subjects divided into three groups: 20 healthy normal individuals as a control group, 20 patients with stable angina (atherosclerotic plaque), and 20 patients with myocardial infarction. Fasting blood samples were withdrawn from all subjects and serum was prepared. SAA, resistin, and adiponectin levels were quantitatively measured by enzyme-linked immunosorbent assay (ELISA).

Results

The SAA level was significantly higher in both stable angina and the acute myocardial infarction group than the control group (2.7179 ± 0.44501 mg/L) and the serum resistin level was significantly higher (p-value = 0.0) in the stable angina (8.368 ± 1.633 ng/ml) and the acute myocardial infarction (13.606 ± 2.067 ng/ml) groups (p-value= 0.0) than the control group. (2.4272±1.25210 ng/ml). Moreover, resistin levels in stable angina when compared to the AMI showed a significant difference between them (p-value = 0.0) while adiponectin was significantly lower in the acute myocardial infarction group. (6.641±2.6011 µg/mL, p-value = 0.019) than its level in the control group (11.873±1.798 µg/mL). While the adiponectin level showed no significant differences between stable angina in comparison to the AMI.

Conclusion

SAA can be used as a confirmatory marker for stable angina and a diagnostic tool for AMI patients. Both SAA and resistin may participate in the atherosclerosis process as an effectors molecule of inflammatory reactions. For adiponectin, we concluded that it has the antiatherogenic property and its levels were lower in both the stable angina and acute myocardial infarction groups.

## Introduction

Atherosclerosis is a progressive disease characterized by the accumulation of lipids and fibrous elements in the arteries, which lead to thrombus formation [1]. It is a lesion made of three compartments: the first is cellular, composed of smooth muscle cells and macrophages, the second compartment is connective tissue and extracellular lipid, and the third compartment is an intracellular lipid that is found within the macrophages. It is developed as an inflammatory stimulus, subsequent to many cytokines and the release of inflammatory markers [2].

The pro-inflammatory protein serum amyloid A (SAA) is a sensitive marker of acute inflammatory reactions released in atherosclerosis. It is able to impair the anti-oxidative properties of high-density lipoprotein (HDL), and this contributes to oxidative stress that occurs in atherosclerosis. So, SAA is considered predictive for atherosclerosis [3].

Adiponectin (an adipocyte-derived cytokine) has potential anti-atherogenic and anti-inflammatory properties. It involves a defense mechanism against thrombus formation. When the endothelium of the vessel is damaged, it accumulates in the subintimal space of the arterial wall and interacts with collagen found in the vascular intima [4-5]. In the Pischon T study (2004), it was found that a high plasma adiponectin concentration is associated with a lower risk of myocardial infarction (MI). This relationship can be only explained by the difference in blood lipids and it is independent of inflammation.

Resistin is an adipokine having a role in the progression of atherosclerosis and its elevation predicts myocardial infarction. It increases the expression of pro-atherogenic molecules as endothelin 1 (ET-1), vascular cell adhesion molecule (V-CAM), and monocyte chemo-attractant chemokine-1 (MCP-1), and it down-regulates anti-atherogenic molecules like tumor necrosis factor (TNF)-receptor-associated factor 3 (TRAF-3). Resistin is expressed by macrophages infiltrated into the atheromatous plaque, which suggests that resistin may act as a modulator for macrophages-to-foam cell transformation [6].

MI is defined as myocardial cell death due to prolonged ischemia. Cell death is categorized as coagulation and/or contraction band necrosis, which usually evolves through oncosis but can result in a lesser degree of apoptosis of the cardiac muscle [7].

SAA was measured in patients with myocardial infarction and was found to be increased in those patients. Its concentration peaks between two and four days after myocardial infarction and decreases later. There was a significant association between maximum SAA and maximum creatine kinase concentrations by using correlation coefficients [8]. The concentration of SAA was found to be correlated to the amount of damaged tissue [9].

Resistin impairs glucose uptake in cardiomyocytes by a mechanism that involves altered vesicles trafficking. There was a positive relationship between resistin and MI [10]. In this study, it was found that the serum resistin levels were significantly increased in patients that presented with acute MI in relation to the control group [11]. The aim of this study is to evaluate the changes in the inflammatory mediator’s SAA, adiponectin, and resistin in the serum of patients with stable angina and acute myocardial infarction and correlate it with troponin-T and creatine kinase-myocardial band (CK-MB).

## Materials and methods

The study was done on 60 subjects divided into three groups: Group I (20 healthy individuals), Group II (20 patients with stable angina), and Group III (20 patients with myocardial infarction). All healthy subjects and patients were males with ages ranging from 40-68 years and selected according to inclusion/exclusion criteria as follows: For the control group, the inclusion criteria were non-smokers, non-diabetic, with no history of an inflammatory disease like gout, lupus erythematosus, bronchial asthma, eczema, arthritis, hepatitis, nephritis, thyroiditis, allergic reactions, multiple sclerosis, rheumatoid arthritis, Crohn’s disease, and ulcerative colitis. And there is no history of cardiac disease or cardiac attack.

For the stable angina group, the inclusion criteria were high blood glucose (diabetics), high blood pressure, high low-density lipoprotein (LDL)-cholesterol, low HDL-cholesterol, elevated C-reactive protein (CRP), slightly elevated troponin-T, history of chest pain (less than 15 minutes, transient usually 3-5 minutes), and a history of recent atherosclerotic plaque and stable angina selected from the cardiology clinic and confirmed by a stress test, angiogram, and ECG.

For the myocardial infarction group, patients were selected from the cardiology unit and the inclusion criteria were the same as that for the stable angina group but with prolonged chest pain (lasting for more than 15 minutes) and a recent cardiac attack confirmed by electrocardiography, blood tests as elevated CK-MB, troponin-T, and myoglobin with an echocardiogram.

Subjects were examined by a cardiologist and they underwent a stress test, angiogram, and ECG to confirm a correct diagnosis. Moreover, routine blood tests were collected as renal function test, hepatic function, lipid profile, troponin T, CK, CK-MB, CRP, fasting blood glucose, and complete blood count (CBC) (was done on a whole blood sample).

A blood sample from the three groups was collected and divided into three tubes (plain tube for serum separation, sodium heparin, and ethylenediaminetetraacetic acid (EDTA) for the plasma needed for the routine chemistry tests).

For all the three analytes, enzyme-linked immunoassay (ELISA) kits (Abcam, Cambridge, UK); for SAA, the human serum amyloid A (SAA) ab100635 ELISA kit was used; for Resistin, the human resistin ELISA kit, ab183364, was used; and for adiponectin, human adiponectin ELISA kit, ab99968, was used. All were quantitatively measured by ELISA for the three groups. The analytes were measured using the instructions by the manufacturers.

Ethical approval

This protocol was approved by the Unit of Biomedical Ethics Research Committee (Reference No.249-14), Faculty of Medicine, King Abdulaziz University, Jeddah, Saudi Arabia. According to our institutional standards, a signed informed consent form was obtained from all participants.

Statistical analysis

Statistical analysis was carried out using Statistical Package for the Social Sciences (SPSS) for Windows, version 23 (IBM Corp., Armonk, NY). The obtained data were presented as means ± standard deviation (SD). Statistical analysis of variance between mean values of different groups was performed using a one-way analysis of variance (ANOVA) followed by a post-hoc test. Differences were considered significant at a p-value of <0.05. The correlation between the two variables was carried out using Pearson's correlation.

## Results

The results of the routine biochemical tests among the studied groups in comparison to the control subjects are presented in Table 1. The levels of troponin-T, CK-MB, SAA, resistin, and adiponectin in the studied groups are presented in Table 2.

**Table 1:** Routine laboratory results among studied groups in comparison to control ALT: Alanine transaminase, AST: Aspartate aminotransferase, CK: Creatine kinase, CK-MB: CK-myocardial band, CRP: C-reactive protein, HDL: High-density lipoprotein, Hba1c: Hemoglobin a1c, LD: Lactate dehydrogenase, LDL; low-density lipoprotein, Na: Sodium, K: Potassium, TG: Triglycerides *P1 value is the significant difference between the control group and stable angina. *P2 value the significant difference between the control group and acute myocardial infarction (AMI).

Test	Control n=20 mean ± SD	Stable angina n=20 mean ± SD	Acute myocardial infarction n=20 mean ± SD	P1 value	P2 value
CK (IU/L)	165.05 ± 94.55	244.8 ± 304.2	270.8 ± 205.43	0.00*	0.00*
AST (U/L)	27.6 ± 3.6	32.77 ± 26.7	39.6 ± 46.6	0.001*	0.002*
Total cholesterol (mmol/L)	4.00 ± 1.1	4.069 ± 1.036	4.04 ± 1.108	0.001*	0.002*
HDL (mmol/L)	1.2035 ± 0.3588	1.07 ± 0.212	1.045 ± 0.239	0.008*	0.015*
LDL (mmol/L)	3.3615 ± 0.632	2.695 ± 0.855	2.745 ± 1.37	0.003*	0.01*
TG (mmol/L)	1.152 ± 0.986	1.422 ± -0.572	1.617 ± 1.132	0.015*	0.001*
Glucose (mmol/L)	5.543 ± 3.8	7.245 ± 2.4	8.45 ± 2.92	0.002*	0.005*
Hba1c (%)	4 ± 2.23	7.14 ± 2.004	7.448 ± 1.853	0.01*	0.003*
Troponin T (µg/L)	0.02 ± 0.01	4.165 ± 1.22	14.665 ± 3.325	0.00*	0.00*
CKMB (µg/L)	1.67 ± 1.28	15.335 ± 2.05	42.89 ± 4.94	0.003*	0.001*
ALT (U/L)	43.86 ± 3.34	34.55 ± 3.5	57.2 ± 3.2	0.03*	0.001*
LD (U/L)	147.4 ± 0.674	245.2 ± 35.2	434 ± 48.3	0.001*	0.05*
NA (mmol/L)	139.98 ± 0.026	138 ± 0.521	135 ± 0.612	0.0*	0.01*
K (mmol/L)	4.28 ± 0.042	4.41 ± 0.011	4.2 ± 0.012	0.0*	0.01*
Urea (mmol/L)	4.54 ± 0.322	6.23 ± 1.22	8.66 ± 1.53	0.001*	0.005*
CRP (mg/L)	2.01 ± 0.23	5.13 ± 1.067	18.9 ± 2.7	0.003*	0.001*

**Table 2:** Serum amyloid A, resistin, and adiponectin results in the studied groups and their p-values * Statistically significant difference (P-value <0.05)

Studied groups	Serum amyliod A (mg/L) mean ± SD	Resistin (ng/mL) mean ± SD	Adiponectin (µg/mL) mean ± SD
Control	2.7179 ± 0.445	2.427 ± 1.252	11.873 ± 1.798
Stable Angina	23.0898 ± 2.88	8.368 ± 1.633	8.615 ± 0.192
Acute myocardial infarction	11.873 ± 1.798	11.873 ± 1.798	6.641 ± 2.601
P-value			
Between the studied groups	<0.001*	<0.001*	0.059
Control vs Stable angina	<0.001*	<0.001*	0.138
Control vs Acute myocardial infarction	<0.001*	<0.001*	0.019*
Stable angina vs Acute myocardial infarction	<0.001*	<0.001*	0.366

Serum amyloid A, resistin, and adiponectin in the studied groups

The SAA level in the serum of the control group was 2.717 ± 0.445 mg/L. In the stable angina group, it was 23.089 ± 2.88 mg/L while in the AMI group, it was 41.555 ± 2.24 mg/L. The level of SAA was significantly higher in the stable angina (P-value=0.0) and acute myocardial infarction (P-value = 0.0) groups as compared to its level in the control group. There was a statistically significant difference between groups as determined by one-way analysis of variance (ANOVA) (P-value=0.0) (Table 2).

The resistin level in the serum of the control group was 2.427±1.252 ng/ml, its level in the stable angina group was 8.368 ± 1.633 ng/ml and in the AMI group, it was 13.606 ± 2.067 ng/ml. Resistin levels were significantly higher in both the stable angina (P-value=0.0) and acute myocardial infarction AMI (P-value=0.0) groups as compared to the control group. There was a statistically significant difference between groups as determined by one-way ANOVA (P-value=0.0) (Table 2).

The level of adiponectin in the serum of the control group was 11.873±1.798 µg/m while in the stable angina group, it was 8.615±0.192 µg/m and in the AMI group was 6.641±2.601 µg/mL. Adiponectin was insignificantly lower in the stable angina group (P-value=0.138) and was significantly lower in the AMI group (P-value=0.019) than the control group. There was no statistically significant difference between groups as determined by one-way ANOVA (P-value=0.059) (Table 2).

Serum amyloid A, resistin, and adiponectin in the stable angina vs. acute myocardial infarction group

The level of SAA was significantly higher in the AMI group (41.555 ±2.24 mg/L) than its level in the stable angina group (23.089 ± 2.88mg/L) (P-value=0.0*; Table 2). The level of resistin was significantly higher in the AMI group (13.606 ± 2.067 ng/ml) than its level in the stable angina group (8.368 ± 1.633 ng/ml) (P-value=0.0*; Table 2). The level of adiponectin was insignificantly lower in the AMI group (6.641 ± 2.601 µg/ml) than its level in the stable angina group (8.615 ± 0.192 µg/ml) (P-value = 0.366; Table 2).

Correlation between SAA and resistin in the stable angina group

**Figure 1:**
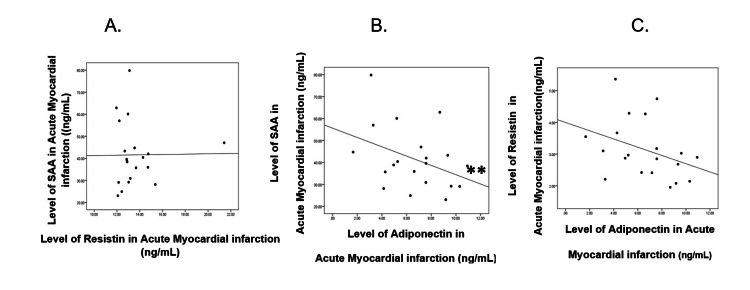
Correlation between SAA, resistin, and adiponectin in the acute myocardial infarction group SAA: serum amyloid A

A weak positive correlation was found between SAA and resistin in the stable angina group. The correlation value r = 0.023. (Positive means that when the variable increases in value, the second variable increases its value. The nearer the value to zero, the weaker the relationship; the nearer to 1; the stronger the relationship). There was no statistically significant correlation between the two variables (P-value=0.461; significant P-value < 0.05) (Figure 1, panel A).

Correlation between SAA and adiponectin in the stable angina group

There was a weak negative correlation found between SAA and adiponectin in the stable angina group. The correlation value r = - 0.255. (Negative means that when a variable increases in value, the second variable decreases in value). There was no statistically significant correlation between the two variables (P=0.139) (Figure 1, panel B).

Correlation between resistin and adiponectin in the stable angina group

A weak negative correlation was found between resistin and adiponectin in the stable angina group. The correlation value r = - 0.162. There was no statistically significant correlation between the two variables (P=0.496) (Figure 1, panel C).

Correlation between SAA and resistin in the AMI group

**Figure 2:**
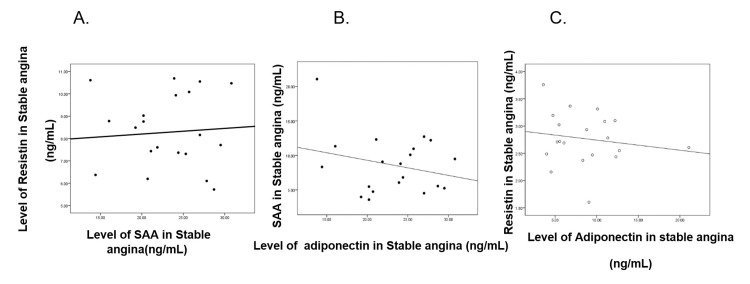
Correlation between SAA, resistin, and adiponectin in the stable angina group

A weak positive correlation was found between SAA and resistin in the AMI group.The correlation value r = 0.011. There was no statistically significant correlation between the two variables (P-value=0.481) (Figure 2, panel A).

Correlation between SAA and adiponectin in the AMI group

A negative correlation was found between SAA and adiponectin in the AMI group. The correlation value r = -0.387. There was a statistically significant correlation between the two variables (P=0.046) (Figure 2, panel B).

Correlation between resistin and adiponectin in the AMI group

A negative correlation was found between resistin and adiponectin in the AMI group. The correlation value r = -0.363. There was no statistically significant correlation between the two variables (P-value=0.115) (Figure 2, panel C).

Correlation between SAA and troponin-T in the stable angina group

**Figure 3:**
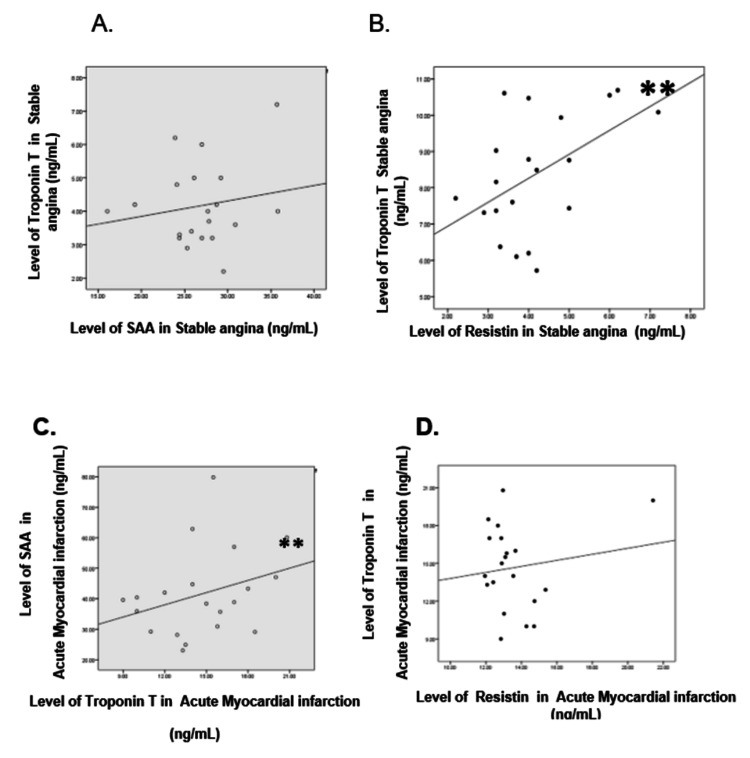
Correlation of troponin-T with SAA and resistin in the stable angina group and in the AMI group AMI: acute myocardial infarction

A weak positive correlation was found between SAA and troponin-T. The correlation value r = 0.173, There was no statistically significant correlation between the two variables (P=0.233) (Figure 3, panel A).

Correlation between resistin and troponin-T in the stable angina group

A positive correlation was found between resistin and troponin-T. The correlation value r = 0.498. There was a statistically significant correlation between the two variables (P=0.013) (Figure 3, panel B).

Correlation between SAA and troponin-T in the acute myocardial infarction group

A moderate positive correlation was found between SAA and troponin-T. The correlation value r = 0.512. There was a statistically significant correlation between the two variables (p=0.0) (Figure 3, panel C).

Correlation between resistin and troponin-T in the acute myocardial infarction group

A weak positive correlation was found between resistin and troponin-T. The correlation value r = 0.149. There was no statistically significant correlation between the two variables (P=0.265) (Figure 3, panel D).

Correlation between SAA and CK-MB in the stable angina group

**Figure 4:**
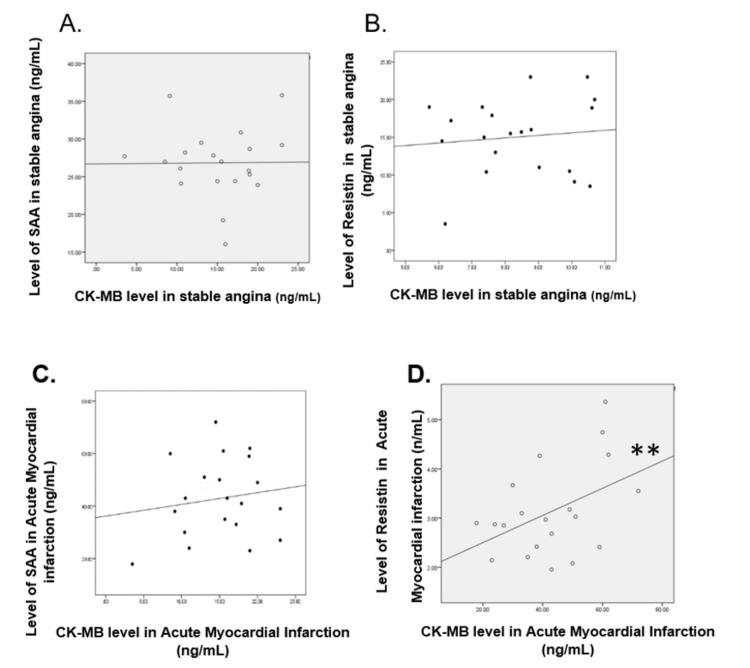
Correlation of CK-MB with SAA, resistin, and adiponectin CK-MB: creatine kinase-myocardial band; SAA: serum amyloid A

A weak positive correlation was found between SAA and CK-MB in the stable angina group. The correlation value r =0.011. There was no statistically significant correlation between the two variables (P=0.482) (Figure 4, panel A).

Correlation between resistin and CK-MB in the stable angina group

A weak positive correlation was found between resistin and CK-MB in the stable angina group. The correlation value r = 0.108. There was no statistically significant correlation between the two variables (P=0.325) (Figure 4, panel B).

Correlation between CK-MB and SAA in the acute myocardial infarction group

A weak positive correlation was found between SAA and CK-MB in the AMI group. The correlation value r = 0.151. There was no statistically significant correlation between the two variables (P=0.262) (Figure 4, panel C).

Correlation between CK-MB and resistin in the acute myocardial infarction group

A moderate positive correlation was found between resistin and CK-MB in the AMI group. The correlation value r = 0.444. There was a statistically significant correlation between the two variables (P=0.025) (Figure 4, panel D).

## Discussion

In our study, we evaluated the changes in inflammatory mediator SAA. Serum amyloid A is an acute phase reactant whose levels are elevated suddenly as the body responds to many injuries like trauma, infection, and inflammation. Its site of expression proved to be the liver [12-13].

Also, extrahepatic SAA expression was found in many tissues and the cells of atherosclerotic plaques. Its expression in the epithelial compartment of many tissues, including the breast, stomach, small and large intestines, prostate, lung, pancreas, kidneys, tonsils, thyroid, pituitary, placenta, skin epidermis, and brain neurons, and was observed in lymphocytes, plasma cells, and endothelial cells [14-15]. Also, it is found in atherosclerotic plaque combined with the lipid compartment in the plaque. It has a biological role in the formation of atherosclerosis [16]. In the acute phase, SAA is synthesized by the liver and transported combined with HDL (apoB-containing lipoproteins) to the atherosclerotic plaque. It's found that it enhances the formation of plaque and increases atherosclerosis events [17]. In another study, It was proven that SAA is a good prognostic marker for stable angina and AMI patients and has a wide dynamic range so it is more useful to be used in routine practice [18].

In addition, another study found that SAA correlates to the size of the tissue injury that happened within the myocardial infarction and responded faster than CRP. So, SAA level elevation leads to acceleration of the atherosclerotic events [17-19].

Jhonson BD et al. [20] and Ridker PM et al. [21] proved that SAA increases will predict cardiovascular thrombosis better than CRP. SAA has also been proved as being a biomarker of cerebrovascular disease and carotid artery intima-media thickness, which is an early stimulus of atherosclerosis [22].

In our study, the level of SAA in the stable angina group was 23.0898 ± 2.88 mg/L, and in the acute myocardial group, it was found to be 41.555± 2.24 mg/L. Many studies were compatible with our finding that the SAA level was significantly higher in both the stable angina and acute myocardial infarction groups than the control group (2.7179 ± 0.44501 mg/L). Also, we compared the SAA level in stable angina versus the SAA level in AMI, and there was a significant difference between them (P-value=0.0). According to these results, we conclude that serum amyloid A protein is the best marker of both stable angina and AMI. Also, SAA is an acute-phase protein investigated for the early prediction of angina [9,17,19].

The higher level of SAA in the stable angina and AMI groups than the control group is due to the release of SAA in the inflammatory condition as atherosclerosis. Also, SAA is bound to HDL (apoB-containing lipoproteins) and changes its protective role, which may enhance the formation of the atherosclerotic plaque, which increases atherosclerosis [17].

We have been detecting SAA in the control group because SAA was produced in normal human extrahepatic tissues [14].

Resistin belongs to a newly discovered protein family and was found in inflammatory zones. It is expressed only in white adipose tissue and is also detected in peripheral blood monocytes, which suggests its role in inflammatory processes [23]. It is an inflammatory cytokine that is found to be involved in atherosclerosis progression by increasing lipid accumulation. The resistin has an inflammatory property, as it acts as a modulator for the macrophage to foam cell formation by increasing the expression of macrophage scavenger receptor (SR-A) and CD36 [24-25].

Also, it was found that resistin increases the uptake of oxidized LDL. The oxidized LDL dysregulates vascular endothelial function by increasing the production of superoxide and decreases endothelial nitric oxide synthase, therefore decreasing the nitric oxide (NO) level. A study found that nitric oxide protects against atherogenesis by preventing smooth muscle cell proliferation and leukocyte adhesion. So, when resistin decreases the NO level, it will enhance atherosclerotic plaque formation [26-27].

Although resistin was found in normal individuals with inflammatory processes and in many tissues like the placenta, skeletal muscle, small intestine, spleen, stomach, thymus, thyroid gland, and uterus. Its expression is in white adipose tissue in macrophages [28-29].

In the Reilly MP et al. study, it was found that the resistin level was higher in stable angina and in acute myocardial infarction groups. The levels of resistin were compared to inflammatory markers and were prognostic of coronary atherosclerosis, independent of CRP [29]. Also in the Ibrahim AE study, the resistin level was found higher in acute myocardial infarction patients than in a healthy normal group [30].

In our study, we found that the serum resistin level was significantly higher (P-value=0.0) in the stable angina (8.368 ± 1.633 ng/ml) and acute myocardial infarction (13.606 ± 2.067 ng/ml) groups (P-value=0.0) than the control group (2.4272±1.25210 ng/ml). We also compared the level of resistin in stable angina versus AMI; there was a significant difference between them (P-value=0.0).

The higher resistin levels in stable angina and AMI maybe because resistin is involved in thrombosis through its regulation of the endothelial nitric oxide synthase enzyme that plays a significant role in platelet aggregation, which leads to plaque formation [31]. Also, resistin combined with atherosclerosis by endothelial cells activation leads to endothelial dysfunction and stimulates multiple pro-atherosclerotic processes [32-33]. So, resistin level elevation increases atherosclerosis events.

The last parameter we focused on in our study was adiponectin, which is an adipocytokine that suppresses atherogenesis by inhibiting monocyte adherence, reducing phagocytic activity, and suppressing lipoprotein accumulation in the vessel wall. It lowers the injury of endothelial cells and stimulates nitric oxide release from endothelial cells in the vessels [34].

In the Kato H et al. and Ekmekci H and Ekmekci OB studies, it was found that low levels of adiponectin lead to thrombus formation and platelet aggregation so it increases acute myocardial infarction occurrence [34-35]. So, adiponectin is considered to be an endogenous antithrombotic factor. Also, in the Pischon T et al. study, it was found that adiponectin levels were significantly lower in acute myocardial infarction patients than in a control group [36].

In our study, we found that adiponectin was insignificantly lower in the stable angina group (8.615±.4.1925 µg/mL, P-value=0.138) and was significantly lower in the acute myocardial infarction group (6.641±2.6011 µg/mL, P-value=0.019) than its level in the control group (11.873±1.798 µg/mL). Also, we compared the adiponectin level in stable angina versus the AMI; there were no significant differences between them.

We found that there was an insignificant lower level of adiponectin in stable angina compared with the control group. This finding was compatible with the Pilz et al. study [37]. Also, was found that there were significantly lower levels of adiponectin in the AMI compared to the control group. This finding was compatible with the Nakamura et al. study [38]. 

The insignificant low levels of adiponectin in the stable angina group may be due to the incomplete formation of the complex lesion of atherosclerotic plaque. The significant low levels of adiponectin in the AMI group would be due to the complete formation of this complex.

Plasma adiponectin is decreased in AMI patients and in stable angina patients because adiponectin was proved to be anti-atherogenic and anti-inflammatory effectors. Also, it inhibits the lipid from accumulating in human monocytes and inhibits macrophages from cell transformation by suppressing the TNF-α-induced monocyte adhesion and the expression of endothelial leukocyte adhesion molecule-1 (E-selectin), vascular cell adhesion molecule -1 (VCAM-1), and intracellular adhesion molecule-1 (ICAM -1) on the endothelium. These adhesion molecules' expression plays an important role to regulate atherosclerosis [39-40].

In our study, we found that SAA and resistin have a positive correlation in stable angina (r = 0.023) and in AMI (r = 0.011), with no significant differences, respectively (P-value=0.461) and (P-value=0.481).

Also, in our study, we noticed that SAA has an insignificant negative correlation with adiponectin in the stable angina (r = - 0.255, P-value=0.139) while in the AMI group, SAA has a significant negative correlation (r = - 0.387, P-value=0.046).

We also noticed that SAA has an insignificant positive correlation with troponin-T levels in the stable angina group (r = 0.173, P-value=0.233). While SAA has a significant positive correlation with troponin-T levels in the AMI group (r = 0.512, P-value=0.03). In stable angina, the SAA level was (23.0898 ± 2.88) and the troponin-T level was 4.165 ± 1.22. While in AMI, the SAA level was 41.555 ± 2.24 and the troponin-T level was 14.665 ± 3.325. Many studies proved the positive correlation between SAA and troponin-T in stable angina and AMI patients.

We found that resistin has a significant positive correlation with troponin-T level in the stable angina group (r = 0.498, P-value=0.013), and an insignificant positive correlation in the AMI group (r = 0.149, P-value=0.265).

In stable angina, the resistin level was 8.368 ± 1.633 and the troponin-T level was 4.165 ± 1.22 while in AMI, the resistin level was 13.606 ± 2.067 and the troponin-T level was 14.665 ± 3.325. Many studies proved the positive correlation between resistin and troponin-T in stable angina and AMI patients [41].

Also, SAA has an insignificant positive correlation with the CK-MB level in both the stable angina (r = 0.011, P-value=0.482) and AMI groups (r =0.151, P-value=0.262). In stable angina, the SAA level was 23.0898 ± 2.88 and the CK-MB level was 15.035± 2.05 while in AMI, the SAA level was 41.555 ± 2.24 and the CK-MB level was 42.89 ± 4.94. Many studies proved the positive correlation between SAA and CK-MB in stable angina and AMI patients [42-43].

We also noticed that resistin has an insignificant positive correlation with the CK-MB level in the stable angina group (r = 0.108, P-value=0.325) and a significant positive correlation in the AMI group (r = 0.444, P-value=0.025). In stable angina, the resistin level was 8.368 ± 1.633 and the CK-MB level was 15.035± 2.05 while in AMI, the resistin level was 13.606 ± 2.067 and the CK-MB level was 42.89 ± 4.94. Many studies proved the positive correlation between resistin and CK-MB in stable angina and AMI patients [41].

Limitation

The incubation time in ELISA kits was too long so SAA may not be suitable for the rapid diagnosis of AMI by this method. In addition, the SAA kit was expensive and it was challenging to follow up with patients. Finally, the sample size was small and no females were included in the study.

## Conclusions

From our study, we concluded that SAA was strongly connected with the activity of the disease and can be used as a confirmatory marker for stable angina and a diagnostic tool for AMI patients. SAA levels increase with cardiovascular disease events. We also found that the levels of resistin in stable angina and in acute myocardial infarction were correlated with markers of inflammation and are predictive of AMI. So, resistin may be used as a diagnostic biomarker for both groups and may represent a novel link between metabolic signals, inflammation, and atherosclerosis. Moreover, both SAA and resistin have a positive correlation with troponin-T as well as CK-MB, which indicates that the patient has or will have atherosclerotic plaque formation. Both SAA and resistin may participate in the atherosclerosis process as an effector molecule of inflammatory reactions. For adiponectin, we concluded that it has the anti-atherogenic property and its levels were lower in both the stable angina and acute myocardial infarction groups than the control group, suggesting that its lowered level may indicate the severity of CVD and the thrombus complex formation. Adiponectin measurement may be helpful in patients that are at risk of AMI.

We recommend that further study is needed on SAA and resistin by taking samples during an AMI or after the AMI and monitoring and evaluating the levels of each parameter at the proper time. The study should be of a larger sample size and must include female participants.
